# An Investigation into the Prevention of Turnover of Medical Interpreters

**DOI:** 10.14789/jmj.JMJ24-0007-OA

**Published:** 2024-07-26

**Authors:** YUKARI ASAI, JIE HE, SAWAKO SUZUTA, JINGHUA YANG, FRANCOIS NIYONSABA, NAOKO ONO, AI IKEDA

**Affiliations:** 1 Department of Public Health, Juntendo University Graduate School of Medicine, Tokyo, Japan; 1 Department of Public Health, Juntendo University Graduate School of Medicine, Tokyo, Japan; 2 Medical Interpreting, Juntendo University Graduate School of Medicine, Tokyo, Japan; 2 Medical Interpreting, Juntendo University Graduate School of Medicine, Tokyo, Japan; 3 Faculty of International Liberal Arts, Juntendo University, Tokyo, Japan; 3 Faculty of International Liberal Arts, Juntendo University, Tokyo, Japan; 4 Atopy/Allergy Research Center, Juntendo University Graduate School of Medicine, Tokyo, Japan; 4 Atopy/Allergy Research Center, Juntendo University Graduate School of Medicine, Tokyo, Japan

**Keywords:** stress coping, mental stress, social support, professional carrier maturity, job continuity intentions

## Abstract

**Objective:**

Since there have been no studies for the prevention of job turnover among medical interpreters, this study examined the effects of social support, professional career maturity and stress coping on their attitudes toward job continuity intentions.

**Design:**

A cross-sectional study was conducted to examine the relationships between social support, professional career maturity, stress coping and job continuity intentions.

**Methods:**

Stress coping was measured by using a simplified stress coping scale (with 9 items and 1 factor structure). Social support was measured by defining the interpreters who answered “Yes” for “I have someone to talk to when I feel emotional stress”. Professional carrier maturity was assessed by using, 12 career-related items. We defined those interpreters who responded “No” to “Have you ever wanted to quit medical interpreting due to emotional stress?” were to have job continuity intentions.

**Results:**

The present study indicated that 14 (25.5%) of the interpreters did not intend to continue their occupation because of their psychological stresses. Compared to interpreters without social support, the odds ratio of job continuity intentions was 4.39 (95% confidence interval [CI] : 1.13-18.3) for those with social support. Moreover, in comparison with the interpreters with low professional career maturity, the odds ratio of job continuity intentions was significantly higher for those with high professional career maturity (odds ratio [OR] = 4.35; 95%CI: 1.12-21.8). However, there was an association found for stress coping.

**Conclusions:**

Strengthening social support and helping professional career development were the important factors for medical interpreters to be able to continue their careers.

## Introduction

As medical tourism and inbound activities (i.e., tourism, travel, and business) are likely to increase rapidly when normality is restored with regards to COVID-19^1)^, the demand for medical interpreters is also expected to rise^2)^. For instance, approximately 2.88 million foreigners (2.2% of the total population at the end of FY2020) live in Japan^3)^. The demand for medical interpreters has been increasing^4)^. And due to language barriers and differences in culture, religion, and lifestyle, only 2.2% of the medical institutions were able to provide services to the required department for foreign patients^5)^. This reality implies that there is virtually no medical support system for non-Japanese residents. Moreover, benefits in relation to improved health habits from continuous usage of medical interpreters is estimated to be worth roughly 5.8 billion yen by the MHLW^6)^. Yet, the reality is that the medical interpretation field in our country is very much behind compared to Western countries^7, 8)^. As a result, a shortage of professional medical interpreters seems to be nearing reality^9)^.

Since the medical interpreting system is not standardized in Japan^10-12)^ various organizations and institutions have developed their own systems, yet the recognition of medical interpreters is still low^13)^. Moreover, the salary of a medical interpreter is often said to be at volunteer level and is not sufficient to make a decent living^7, 14)^. For example, a reward, 3,000 Japanese yen (including transportation and income tax) per 3-hour session is paid to medical interpreters by Multicultural Center Kyoto, which is hardly to say an adequate livelihood guarantee. The study also has been pointed out that medical interpreters tended to quit their jobs within five years^15)^.

The current situation clearly highlights the unstable social status of medical interpreters^16)^. In comparison with other conference interpreters, Mizuno and Naito (2015) pointed out the large emotional burden of medical interpreters^17)^. Medical interpreters work in stressful medical-specific situations such as illness, death, injuries, treatments, and lab examinations. However, their duties cannot be fulfilled if they are upset by surgery, emergency care, or the mere sight of blood. In addition, if they empathize too much with the patient, they will experience a lot of mental suffering^17, 18)^. Torikai^19)^ asserts that “Even if the world becomes a multilingual society, medical interpretation will not be handled by automatic interpretation machines”. The rationale for this is that empathy, which medical interpreters can provide, is a function of the mind^20)^. In other words, verbal communication between patients and health care professionals (HCPs) in the clinical environment is extremely delicate and requires a high level of sensitivity, as well as a variety of non-verbal communications^21)^. For medical interpreters to function as professionals while maintaining a certain level of quality, mental and physical health management is considered to be essential. However, there have been no studies on the prevention of turnover as well as stress coping among medical interpreters in Japan^22)^, while there is a report^23)^ of 200 workers who developed mental illnesses and left their jobs due to workplace stress in 2021.

Therefore, it is crucial to investigate how medical interpreters avoid burnout through stress coping and/or social support to prevent them from leaving their jobs to continue their careers.

## Methods

### Subjects

Medical interpreters that were registered with MediPhone (248 interpreters) － a telephone medical interpreter dispatch organization － or the National Association of Medical Interpreters (NAMI; 305 interpreters) were targeted for this study. A total of 55 medical interpreters were enrolled in the present study.

A “Request for Research, Purpose and Significance of the Study” was sent via email to the responsible person at the targeted organization. After obtaining permission, we sent an anonymous questionnaire survey via Google Forms to active medical interpreters registered with the organizations. The explanatory document also included information on the “purpose of the research”, “withdrawal after consent to conduct research”, “handling of personal information” and “consultation service for research subjects”, and checking the consent box was considered to agree with informed consent.

This study was approved by the Juntendo University Graduate School of Medicine Ethics Committee for Medical Research (approval number: Juntendo University Medical Ethics No. E21-0235-M01) and conducted from January 12, 2022, to February 28, 2022 and the questionnaire was conducted in Japanese.

### Measures

The survey took approximately 5 minutes to complete and interpreters were asked stress coping, social support, professional carrier maturity, job continuity intentions and the basic information including age (years), sex (male vs. female), salary (adequate vs. insufficient), native language, and interpreting language. Stress coping was calculated using a simplified (factor-structured) stress coping scale with nine items (Appendix 1)^24)^. And the following four-point scale was used: “1: not at all”, “2: hardly at all”, “3: sometimes”, and “4: often.” Interpreters with a median value of ≥ 27 out of 36 points were defined as having stress coping. Stressful medical interpreting environment was also investigated with the question “In which situation did you feel stressed?” and interpreters responded from answers including “in medical environments”, “psychological problems (anxiety, shock, pressure)”, “responsibility/confidentiality”, and “human relations”. Social support was measured by defining the interpreters who answered “Yes” for “I have someone to talk to when I feel emotional stress” as having social support. For those who answered “Yes” to the above, the following question was also asked: “Who (where) do you consult when you feel emotionally stressed?” This question was asked to induce specific social support by providing multiple answers from nine choices that were categorized into four segments: “1: fellow interpreters”, “2: psychological counseling or others”, “3: family/friends (patients, family, friends)”, and “4: medical personnel (doctors, nurses, clerks)”. To assess professional carrier maturity in the present study, 12 career-related items were taken from the Occupational Career Maturity Measurement Scale (Appendix 2), which was developed by Karino et al.^25)^, with permission of the first author. Occupational career maturity was calculated using the following five-point scale out of 60 points: “1: not applicable”, “2: not very applicable”, “3: undecided”, “4: somewhat applicable”, and “5: very applicable. We used a median value of ≥ 49 to signify a high level of professional carrier maturity. The professional carrier maturity was consisted of three types of questions with three reverse questions (i.e., interest, autonomy, and planning and analysis). Questions 1 to 4 related to interest, 5 to 8 related to autonomy, and 9 to 12 related to planning and analysis. Higher total scores indicated greater professional carrier maturity^26)^.

### Measurement of medical interpreters’ willingness for job continuity intentions

Interpreters who responded “No” to “Have you ever wanted to quit medical interpreting due to emotional stress?” were defined to have job continuity intentions. Interpreters who answered that they have never wanted to quit interpreting due to mental stress were considered to have an awareness of continuity in the profession. We used a question from a previous study^27)^ to confirm their intention to continue in the profession (Appendix 2). The interpreters who answered that they have wanted to quit their job as a medical interpreter were asked in which situations they felt like quitting, with four categories of multiple choice: “1: in the medical environment (consultation room, shock by seeing wounds, anxiety by contacting COVID-19 patients, PTSD)”, “2: psychological problems (mental illness, cancer, emergency, operation, DV and crime victims, death of the patients who interpreted)”, “3: high responsibility and low pay, confidentiality stress”, and “4: human relations with HCPs, with medical interpreters)”.

### Statistical analysis

The difference of proportions and means according to stress coping, social support, and professional carrier maturity categories were tested by Chi- square and T-test. Multivariate logistic regression analysis was used to examine the relationships between job continuity intentions, stress coping, social support, and professional carrier maturity, with adjustments for sex and age. All statistical analyses were performed using the SAS software package (version 9.4, SAS Institute, Cary, NC, USA). All p-values for statistical tests were two-tailed, and values of p < 0.05 were denoted as statistically significant differences.

## Results

The basic characteristics of the interpreters are shown in Appendix 3. The study population consisted of 55 interpreters (7 males and 48 females) registered with a medical interpreting company, with a response rate of 9.9%. The minimum and maximum ages were 32 and 68, respectively, with a mean age of 50.2 years old (SD = 8.76). Most interpreters (36.4%) had been interpreting medical information for “5-10 years”, while 14.5% had been interpreting for “10 years or longer” and 5.5% had been interpreting for “less than 1 year”. Moreover, 60% in interpreters indicated that the salary was insufficient.

Most (80%) of were the native Japanese interpreters, while 20.0% were native speakers of a foreign language. Of the 11 native speakers of foreign languages, 27.3% were native speakers of Chinese and 27.3% were native speakers of Vietnamese, accounting for more than half of the interpreters. There was a total of 16 interpreting languages, with English and Japanese being the most common (31.0%), followed by Chinese and Japanese (16.4%).

In terms of job continuity intentions (JCIs), 25.5% (14 interpreters) answered “I have wanted to quit my job as a medical interpreter due to emotional stress”. The specific situations cited by those who wanted to quit medical interpreting were medical practices (35.7%), psychological problems such as anxiety, shock, and pressure (28.7%), responsibility/confidentiality (21.4%), and human relationships (14.2%).

The highest response regards to social support was from fellows (31%) or psychological counselors or others (31.0%), followed by family and/or friends (28.0%), and medical professionals (10.0%). On the other hand, more than one fourth (27.3%) responded that they have no person or place to consult with when they have psychological stress.

Table 1 shows that the interpreters with social support had high professional carrier maturity (p = 0.02). And among the 15 interpreters without social support, the percentage of the high professional carrier maturity was 62%. It was apparent that the interpreters with social support also had stress coping (92%) and the interpreters without social support were likely to have stress coping (p = 0.007). In terms of professional carrier maturity, interpreters with stress coping seems to have higher professional career maturity (p = 0.05). And the percentage of without stress coping and low professional carrier maturity was the highest (69%) while the percentage of without social support and low professional carrier maturity was 38.0%.

**Table 1:** Background of the medical interpreters

		n	Mean Age (SD)	p value	Medical interpretation history, ％		p value	Sex, %		p value	Salary, ％		p value	Social support, ％		p value	Stress coping, ％		p value	Professional carrier maturity, ％		p value
					< 5 years	≥ 5 years		Male	Female		Ade-quate	Insuffi- cient		With	With out		With	With out		High	Low	
Social support	With	40	50.9 (8.96)	0.33	45.0	55.0	0.38	10.0	90.0	0.38	37.5	62.5	0.7				45.0	55.0	0.007	88.0	12.0	0.02
	With out	15	48.3 (8.11)		60.0	40.0		20.0	80.0		46.7	53.3					87.0	13.0		62.0	38.0	
Stress coping	With	24	48.9 (8.56)	0.34	37.5	62.5	0.13	17.0	83.0	0.44	42.0	58.0	0.88	92.0	8.0	0.007				62.5	37.5	0.06
	With out	31	51.2 (8.92)		58.1	42.9		9.7	90.3		38.7	61.3		58.0	42.0					35.5	64.5	
Professional carrier maturity	High	26	51.0 (8.40)	0.52	54.0	46.0	0.59	15.0	85.0	0.58	38.0	62.0	0.96	85.0	15.0	0.06	58.0	42.0	0.05			
	Low	29	49.5 (9.15)		45.0	55.0		10.0	90.0		41.0	59.0		62.0	38.0		31.0	69.0				

There were significant associations found between job continuity intentions and social support (OR = 4.39; 95%CI: = 1.13-18.31) and job continuity intentions and professional carrier maturity (OR = 4.35; 95%CI: = 1.12-21.85), respectively (Table 2). However, similar results were not observed for stress coping.

**Table 2:** Relationship between social supports, stress coping, professional carrier maturity, and job continuity intentions

		Odds ratio	95％ Confidence Interval	R^2^	p-value
Social support	Crude	4.13	1.13－15.73	4.55	0.03
	Multivariable*	4.39	1.13－18.31	4.46	0.03
Stress coping	Crude	1.04	0.31－3.68	0.005	0.95
	Multivariable*	1.11	0.31－4.13	0.02	0.88
Professional career maturity	Crude	4.69	1.25－23.05	4.56	0.03
	Multivariable*	4.35	1.12－21.85	3.98	0.05

*Adjusted for age and sex.

## Discussion

Recently, surveys of medical interpreters in a wide variety of languages have revealed a variety of conditions involving psychological distress, burden, and stress^28-30)^. For example, a study of interpreters working in the field of refugee care in Germany (164 interpreters) reported significantly higher psychological distress in terms of anxiety and depression, and approximately 7% of those surveyed were positive for post-traumatic stress disorder (PTSD)^31)^. Moreover, Loutan et al. (1999) conducted an anonymous survey of 22 interpreters registered as volunteers with the Red Cross in Geneva^32)^. Five of these interpreters (28%) frequently experienced psychologically difficult emotions while interpreting, while 12 (66%) reported recalling painful memories frequently. According to the Report of the Survey of the Dispatched Medical Interpreters’ Performance in Japan^33)^, the most common stress experienced by medical interpreters is “emotional involvement with the patient” (31%), followed by “stressful due to confidentiality” (22%). In response to this mental stress, strengthening the self-management of medical interpreters has been proposed in recent years^34)^.

Most of the previous studies on stress coping among medical interpreters have been qualitative studies^18, 26, 29)^; instead, this cross-sectional study was a quantitative study based on an online survey. Our study found that interpreters with social support or professional carrier maturity had higher odds of having job continuity intentions. However, we did not find a similar association for stress coping.

Previous studies have shown that many medical interpreters leave the profession due to low pay, high emotional strain, and volunteer status, in contrast to the high levels of skill and responsibility required^35)^.

The results of this study were consistent with previous studies, with more than half (60.0%) reporting that their salary is low^36)^, and more than a quarter (25.5%) stating that they have wanted to quit the medical interpreting profession due to emotional stress^14)^. The results of the survey also agreed with those of previous studies, in that the medical environment was the most common situation in which the interpreters cited wanting to leave their jobs, followed by psychological pressure^32^^-^^33, 37)^.

Social support seems to be a crucial element for the prevention of turnover of medical interpreters. Our results showed that medical interpreters with higher stress coping had a higher percentage of social support (92.0%) compared to those with low stress coping (58.0%), which are consistent with the results of previous studies^16, 18)^. Interpreters with higher professional carrier maturity were more likely to have stress coping. Folkman and Lazarus (1986) examined cognitive appraisal and coping processes in stressful situations and found that coping was strongly related to cognitive appraisal and that forms of coping differed depending on coping options^38)^. This suggests that medical interpreters with high professional carrier maturity and job continuity intentions may have high autonomy^39)^ and are less likely to leave their jobs even if their stress coping is low because of their psychological burdens^40)^. Furthermore, the simplified stress coping scale used in this study contained only item that was classified as cognitive (automatic thinking) coping; all other items were classified as behavioral coping. Although cognitive (automatic thinking) and behavioral coping are considered to be the only effective stress reactions, behavioral coping items such as “enjoyed time with family and friends” and “talked to someone” may not be recognized as coping by certain individuals because these items are not regarded as skills obtained by learning^41)^. Since stress coping is also regarded as self-care^42)^, there may be a need for improved structural regulation of the professional occupation of medical interpreter^6)^.

The results from our study potentially suggest the need for improved social support and working conditions for interpreters. While some studies have focused on stress coping and stress reduction^43-44)^, Nakajima (2006) revealed that social welfare workers who have high social support from supervisors, colleagues, and family and friends are less likely to suffer depression^45)^ and may have a decreased turnover rate. Having high social support can bring a sense of controlling stress^46)^ and intention to work^47)^.

Hence, social support helps to alleviate stress because it may provide instant emotional releases and solutions to problems^48)^.

There were few limitations of this study. Firstly, selection bias may have occurred when selecting the two organizations to be surveyed. The survey was conducted for medical interpreters in Tokyo; therefore, the generalization of our study may be limited. Secondly, this study did not obtain interpreters’ educational background and marital status, which have been shown to be related to stress and professional career maturity in the previous studies^29, 40)^. The results could be confounded by these unmeasured variables. Thirdly, since this is cross-sectional study, causality cannot be shown clearly. Fourthly, interpreters without job continuity intentions were defined as those that answered “Yes” to a single question “I have wanted to quit my job as a medical interpreter due to mental stress.” However, this cannot exclude contradictory human emotion and cognition. In addition, since this survey was conducted during the COVID-19 pandemic, the lower response rate could be due to a reduced demand for medical interpreters because of a burden on medical facilities with policies such as stay homes and avoid the “Three Cs^49)^ (i.e. closed spaces, crowded places, and close-contact settings)”. With regard to this survey, we only know the attributes of the respondents, therefore, the difference in attributes between those who responded to this survey and those who did not cannot be considered. Finally, since this study had a small sample, further research is necessary for more detailed results.

Nonetheless, this is the first quantitative study that examined the impact of social support, stress coping, and professional carrier maturity on job continuity intentions in Japan, as the only a quantitative study has been conducted in Germany^29)^. The results corroborated that professional carrier maturity is related to job continuity intentions of medical interpreters and showed the importance of social support for daily mental stress.

In summary, social support and professional carrier maturity seem to play important roles in the continuity of medical interpreters’ careers. The results of our study suggested that it is important to strengthen social support for mental stress in medical interpreters. In the future, it will be necessary to increase the number of survey participants and conduct a longitudinal study to examine any causal associations between social support and professional carrier maturity. In order to prevent medical interpreters from leaving the profession, it is important to develop a system to enhance their social support and therefore reduce mental stress.

## Funding

No funding was received.

## Author contributions

JH, SS, and JY contributed in discussion for the preparation of the questionnaire and collecting raw data. FN and NO examined the research process and provided various comments on the research. AI analyzed the statistical data and a major contributor in the statistical interpretation. All authors read and approved the final manuscript.

## Conflicts of interest statement

The authors declare that there are no conflicts of interest.

## Supplementary Material

Appendix 1 Stress coping scale (9 items)

Appendix 2 Professional carrier maturity (12 items) and job continuity intentions (1 item) scales

Appendix 3 Basic characteristics of the medical interpreters
