# Supplementary figures and images for: An Investigation into the Prevention of Turnover of Medical Interpreters
Source: Juntendo Iji Zasshi. 2024 Jul 26;70(4):289–99. doi: 10.14789/jmj.JMJ24-0007-OA (PMC11487365; doi:10.14789/jmj.JMJ24-0007-OA)

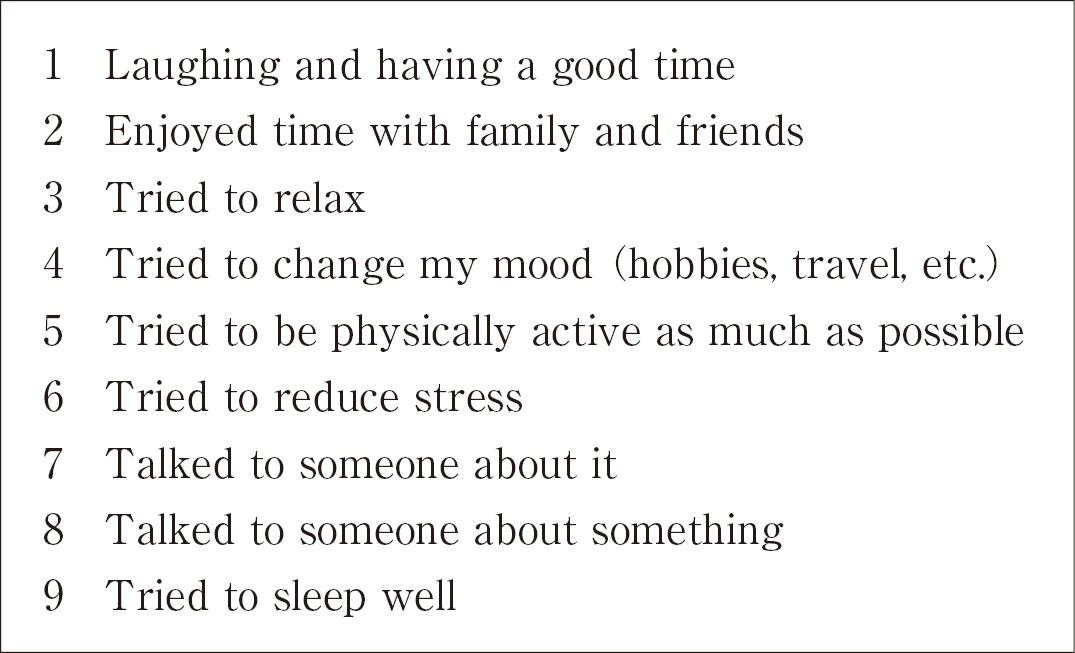

Supplement: Appendix 1 — Stress coping scale (9 items) [file 2188-2126-70-4-0289-s001.jpg]

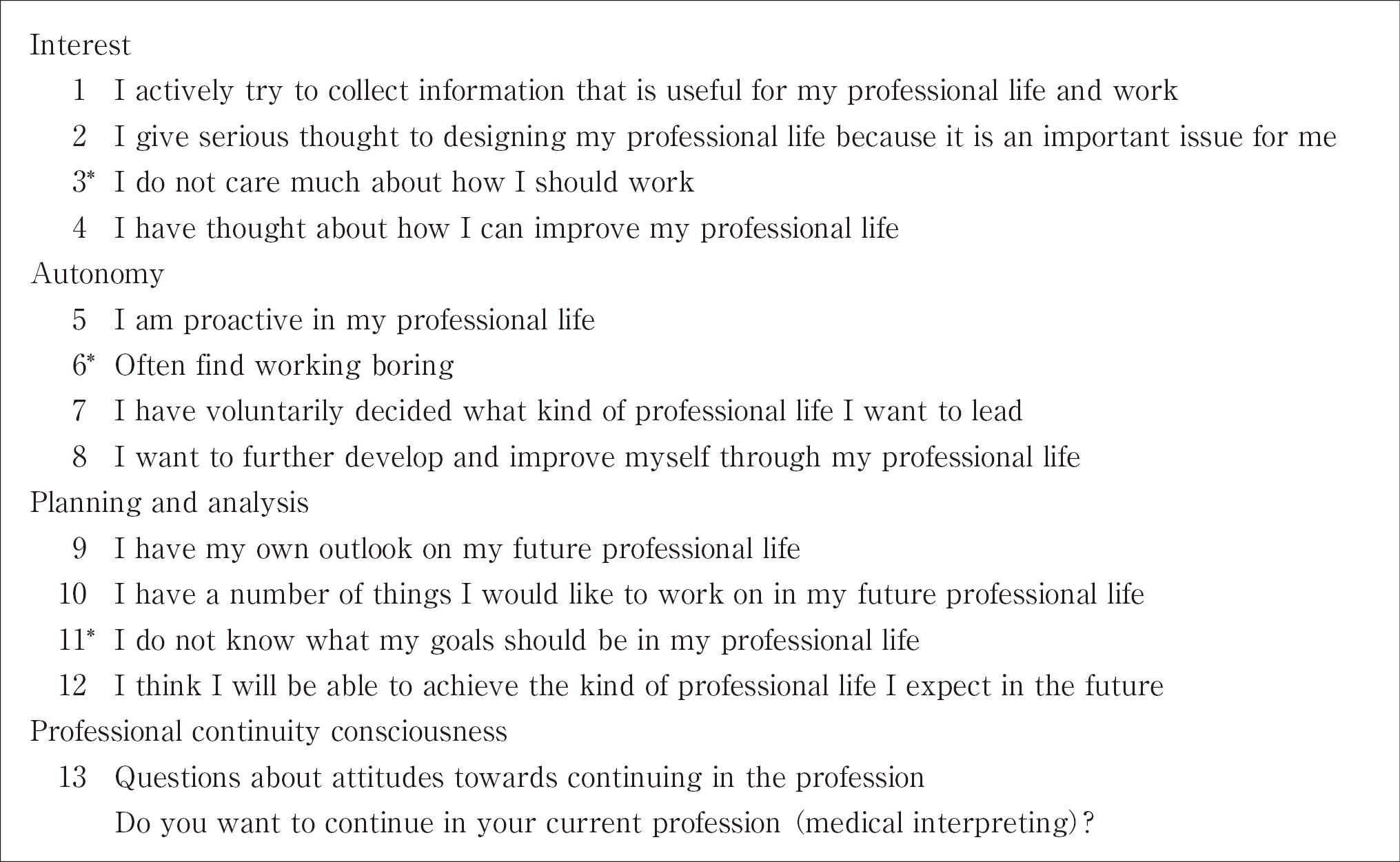

Supplement: Appendix 2 — Professional carrier maturity (12 items) and job continuity intentions (1 item) scales [file 2188-2126-70-4-0289-s002.jpg]

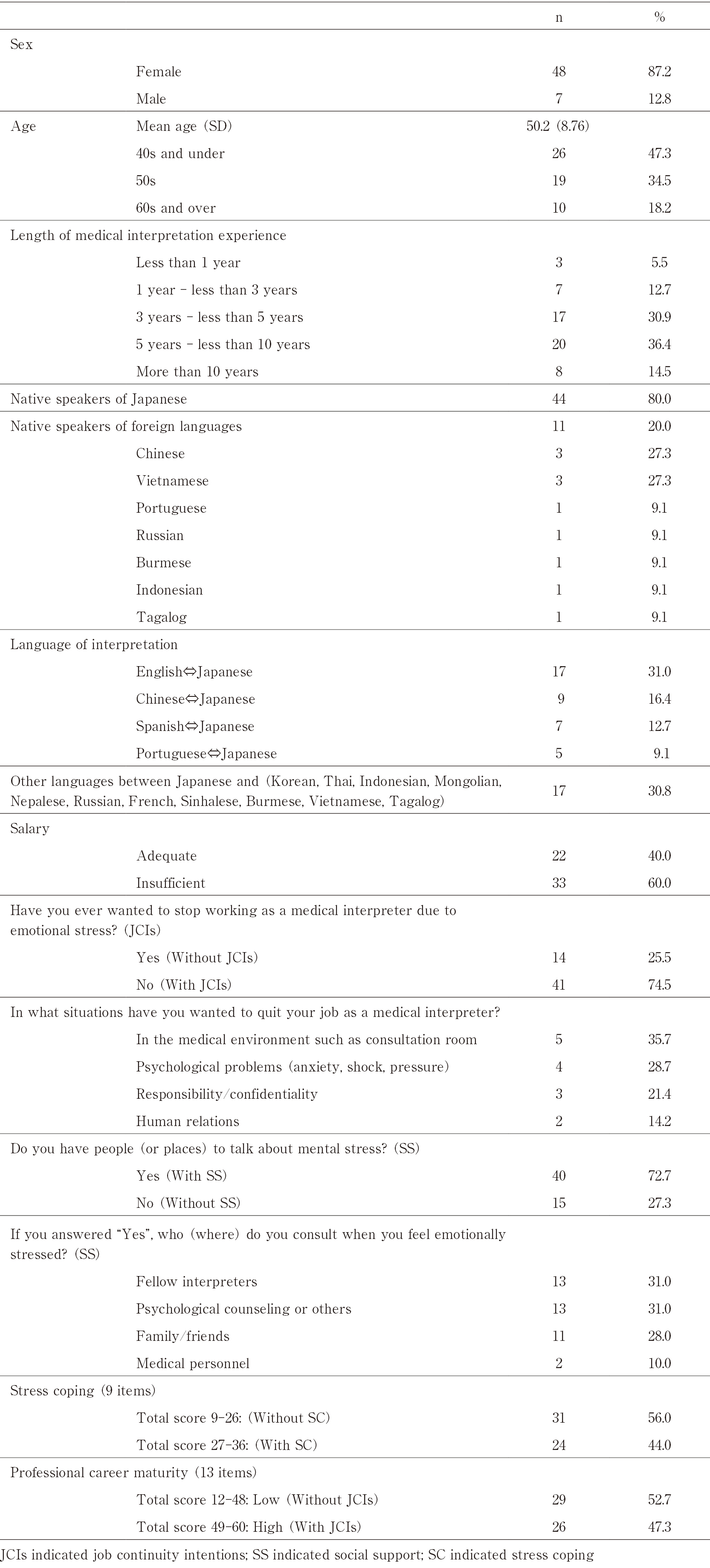

Supplement: Appendix 3 — Basic characteristics of the medical interpreters [file 2188-2126-70-4-0289-s003.jpg]
